# 30-Day Mortality in Candida auris Cases in Tennessee from 2022-2023

**DOI:** 10.1017/ash.2025.332

**Published:** 2025-09-24

**Authors:** Cherlly Bailey, Cody Rocha, Simone Godwin, Melphine Harriott

**Affiliations:** 1 Healthcare-Associated Infections and Antimicrobial Resistance Program, Tennessee Department of Health, Nashville, TN

## Abstract

**Background:** Candida auris, a multi-drug resistant fungal pathogen, was first detected in Tennessee healthcare facilities in 2022. C. auris can colonize a patient’s skin and cause clinical infection. Patients with clinical infections have high mortality, with a wide range of reported rates between 30 – 72%. Here we compare the risk factors associated with 30-day all-cause mortality among colonized and clinical cases in Tennessee. **Method:** Clinical and colonization C. auris case data was obtained from the Tennessee State Public Health Laboratory. Cases with only a skin specimen were classified as colonization, while patients with any other sterile or non-sterile collection site were classified as clinical. Mortality data was obtained through the Tennessee Office of Vital Records and matched with C. auris case information. Risk factors for multi-drug resistant organism acquisition were collected using a REDCap survey completed by facility staff. Chi-square tests were used to compare mortality and risk factor differences. All analyses were conducted in SAS Enterprise Guide v8.3. **Result:** Between 2022 and 2023, 130 out of 418 colonized patients (31.1%) and 33 out of 108 clinical cases (30.1%) died with no significant differences in age. Of the patients that died, 20 (60.6%) with clinical infection and 50 (38.5%) with colonization died within 30 days of specimen collection (p<.05). However, eight patients with clinical infection who died within 30 days of specimen collection were previously colonized. Risk factors associated with C. auris acquisition were available for 55 patients with clinical infection and 120 with colonization. Patients with clinical infection who died within 30 days of specimen collection were more likely to have incontinent urine (p<.05), a draining wound (p<.05), and have a gastric tube placed (p<.05) than those who survived. Patients with colonization who died within 30 days of specimen collection were more likely to have a previous stay in an inpatient rehabilitation facility (p<.01), an ambulatory surgery center (p<.01), and less likely to have a tracheostomy tube placed (p<.05) than those who survived. **Conclusion:** Patients with clinical C. auris infection are more likely to die within 30 days of specimen collection than patients with colonization in Tennessee. However, risk factors associated with C. auris acquisition varied between patients with clinical infection or colonization and are not consistently associated with higher mortality. Clinical teams should emphasize infection prevention and control practices that reduce the risk of invasive infection in colonized patients in all settings, regardless of perceived risk.

